# Green Approach for Synthesis of Silver Nanoparticles with Antimicrobial and Antioxidant Properties from Grapevine Waste Extracts

**DOI:** 10.3390/ijms25084212

**Published:** 2024-04-10

**Authors:** Anda Maria Baroi, Irina Fierascu, Andra-Ionela Ghizdareanu, Bogdan Trica, Toma Fistos, Roxana Ioana Matei (Brazdis), Radu Claudiu Fierascu, Cristina Firinca, Ionela Daniela Sardarescu, Sorin Marius Avramescu

**Affiliations:** 1 National Institute for Research & Development in Chemistry and Petrochemistry–ICECHIM, 202 Spl. Independentei, 060021 Bucharest, Romania; anda.baroi@icechim.ro (A.M.B.); andra.ghizdareanu@icechim.ro (A.-I.G.); bogdan.trica@icechim.ro (B.T.); toma.fistos@icechim.ro (T.F.); roxana.brazdis@icechim.ro (R.I.M.); fierascu.radu@icechim.ro (R.C.F.); cristina.firinca@icechim.ro (C.F.); 2 Faculty of Horticulture, University of Agronomic Sciences and Veterinary Medicine of Bucharest, 59 Marasti Blvd., 011464 Bucharest, Romania; 3 Faculty of Chemical Engineering and Biotechnology, National University of Science and Technology Politehnica Bucharest, 1-7 Gh. Polizu Str., 011061 Bucharest, Romania; ionela.toma93@yahoo.com; 4 Department of Botany and Microbiology, Faculty of Biology, University of Bucharest, 91–95 Spl. Independenței, 050095 Bucharest, Romania; 5 National Research and Development Institute for Biotechnology in Horticulture, Bucharet-Pitesti Str., 117715 Stefanesti, Romania; 6 Department of Inorganic Chemistry, Organic Chemistry, Biochemistry and Catalysis, Faculty of Chemistry, University of Bucharest, 030018 Bucharest, Romania; sorin.avramescu@g.unibuc.ro; 7 Research Centre for Environmental Protection and Waste Management (PROTMED), University of Bucharest, 91–95 Spl. Independenței, Sect. 5, 050107 Bucharest, Romania

**Keywords:** grapevine waste, natural extracts, phytochemicals, green synthesis, antimicrobial activity, characterization

## Abstract

The present work aims to study the possibilities of developing silver nanoparticles using natural extracts of grape pomace wastes originating from the native variety of *Fetească Neagră* 6 Șt. This study focused on investigating the influence of grape pomace extract obtained by two different extraction methods (classical temperature extraction and microwave-assisted extraction) in the phytosynthesis process of metal nanoparticles. The total phenolic content of the extracts was assessed using the spectrophotometric method with the Folin–Ciocâlteu reagent, while the identification and quantification of specific components were conducted through high-performance liquid chromatography with a diode array detector (HPLC-DAD). The obtained nanoparticles were characterized by UV–Vis absorption spectroscopy, X-ray diffraction (XRD), and transmission electron microscopy (TEM), along with assessing their antioxidant and antimicrobial properties against Gram-positive bacteria. The data collected from the experiments indicated that the nanoparticles were formed in a relatively short period of time (96 h) and, for the experimental variant involving the use of a 1:1 ratio (*v*/*v*, grape pomace extract: silver nitrate) for the nanoparticle phytosynthesis, the smallest crystallite sizes (from X-ray diffraction—4.58 nm and 5.14 nm) as well as spherical or semispherical nanoparticles with the lowest average diameters were obtained (19.99–23 nm, from TEM analysis). The phytosynthesis process was shown to enhance the antioxidant properties (determined using the DPPH assay) and the antimicrobial potential (tested against Gram-positive strains) of the nanoparticles, as evidenced by comparing their properties with those of the parent extracts; at the same time, the nanoparticles exhibited a selectivity in action, being active against the *Staphylococcus aureus* strain while presenting no antimicrobial potential against the *Enterococcus faecalis* strain.

## 1. Introduction

Along with the exploitation of vine crops, massive accumulations of viticultural waste such as pomace [1], shoots [2], canes [3], and others are generated worldwide every year, which have a low economic value due to improper management. The most common grapevine waste disposal route includes their storage and burning in open fields, thus becoming a considerable source of greenhouse gas emissions (CO_2_), leading to the contamination of soil and water and endangering fauna [4]. A more promising alternative is represented by a circular bioeconomy approach, in which the wastes are considered as raw materials for other industries (such as pharmaceuticals, environmental applications, agriculture, etc.) and are used to obtain added-value products such as nutraceuticals, cosmeceuticals, sensors, catalysts, etc. This is particularly relevant for viticultural wastes, which are known to be a rich source of biologically active compounds [5,6,7].

Nowadays, when there are a lot of major public health concerns, antimicrobial medications, especially those resulting from emerging fields of research (such as the use of natural sources or nanotechnology) presents a great area of interest, being capable of efficiently managing and averting different infections, particularly those caused by drug-resistant pathogens [7,8]. The majority of antibiotic-resistant strains found in hospitals and that are responsible for most invasive and life-threatening skin and soft tissue infections are Gram-positive bacteria [9], from which *Staphylococcus aureus* is known for its pathogenic nature and tendency to develop multidrug resistance (MDR). This bacterium is more aggressive towards individuals with pre-existent conditions (diabetes, immunocompromised adults) or in neonatal cases. It is capable of producing capsules that result in a unique golden yellow pigment. This pigment serves as both a virulence factor and a defense mechanism against antibiotics, leading to the development of MDR and increased mortality rates [10]. *Enterococcus faecalis* is a Gram-positive bacterium that can survive with or without oxygen and has the ability to cause infections, being frequently encountered in both the oral cavity and gastrointestinal tract of humans [11].

Recently, the scientific community has focused on studying secondary metabolites from viticulture waste as new potential antimicrobial agents [5,7,12]. Grape pomace extract, obtained by a medium-scale ambient temperature (MSAT) system, was assessed for its antibacterial properties against nineteen clinical strain isolates, including some that were resistant to multiple drugs, by Manso et al. [13]. The results indicated that the extract exhibited efficacy against all the tested clinical strains, irrespective of their antibiotic resistance levels. El-Sawi et al. [14] demonstrated that grape stem, seed, and peel extracts exhibited encouraging antibacterial and antifungal properties, and they demonstrated cytotoxic effects on certain cancerous cell lines [14].

Moreover, nanotechnology has emerged as a crucial tool in advancing large-scale technologies across various fields with an emphasis on obtaining different materials with potential for medical applications [8], including the use of silver nanoparticles as potent antimicrobial agents against multidrug-resistant bacterial strains [15] or to control oxidative stress and improve the immune system [16], these aspects being the subject of numerous reviews in the last years. Additionally, innovative methods such as phytosynthesis have made a great contribution to the field of nanotechnology by eliminating the need for hazardous substances [17,18,19]. This not only reduces associated costs but also ensures reproducibility and accelerates the material development process [20]. In phytosynthesis, natural extracts obtained through different extraction techniques are used as reducing agents and surface stabilizers for nanoparticles, thereby minimizing the potential toxicity associated with their use [19,21].

In this context, the present work aimed to study the possibilities of developing silver nanoparticles using natural extracts of grapevine wastes obtained by two different extraction methods. The study was focused on investigating the phytosynthesis process of metallic nanoparticles and the influence of the extract-to-metal-salt ratio along with assessing their antioxidant and antimicrobial properties against Gram-positive strains of clinical interest (*S. aureus* and *E. faecalis*).

## 2. Results

**Figure 1:**
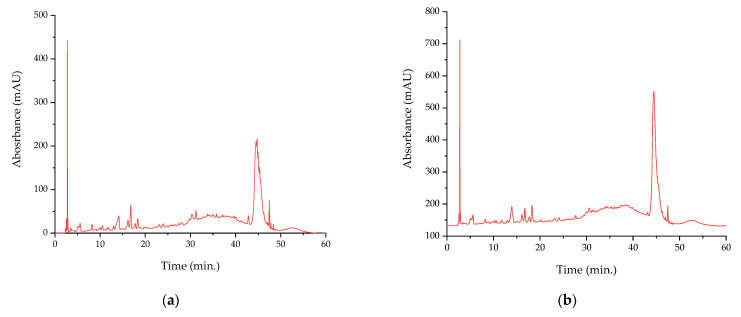
HPLC chromatograms of the analyzed extracts: (**a**) P-T; (**b**) P-Mw; retention times for the identified compounds are presented in Table 1.

The results of the evaluation of the chemical composition of the extracts (in terms of total phenolics and the concentration of target compounds) is presented in Table 1, while the corresponding chromatograms are presented in Figure 1.

**Figure 2:**
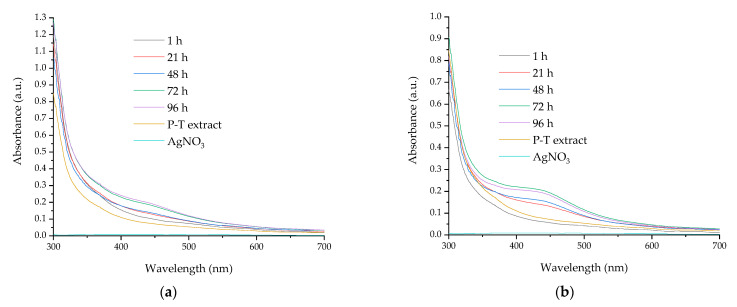
UV–Vis spectra of the nanoparticles synthesized using extract obtained by classical temperature extraction: (**a**) P-T-Ag1 sample; (**b**) P-T-Ag2 sample.

**Figure 3:**
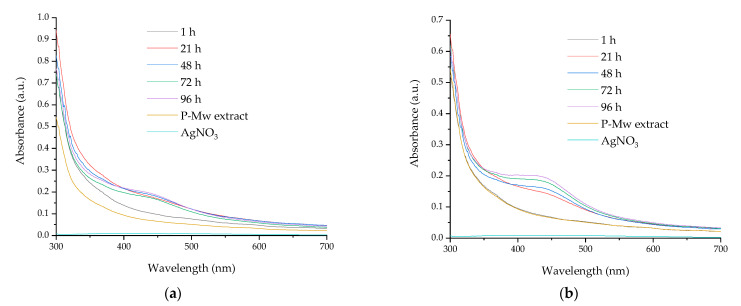
UV–Vis spectra of the nanoparticles synthesized using extract obtained by microwave-assisted extraction: (**a**) P-Mw-Ag1 sample; (**b**) P-Mw-Ag2 sample.

The formation of silver nanoparticles was evaluated using the UV–Vis absorption, with the recorded spectra presenting the specific absorption maxima of the silver nanoparticles. Figure 2 and Figure 3 present the UV–Vis spectra of the phytosynthesized nanoparticles using pomace extract obtained by classical extraction at temperature (P-T) and those developed using pomace extract obtained by microwave-assisted extraction (P-Mw), while Table 2 presents the results collected from the UV–Vis measurements of samples.

**Figure 4:**
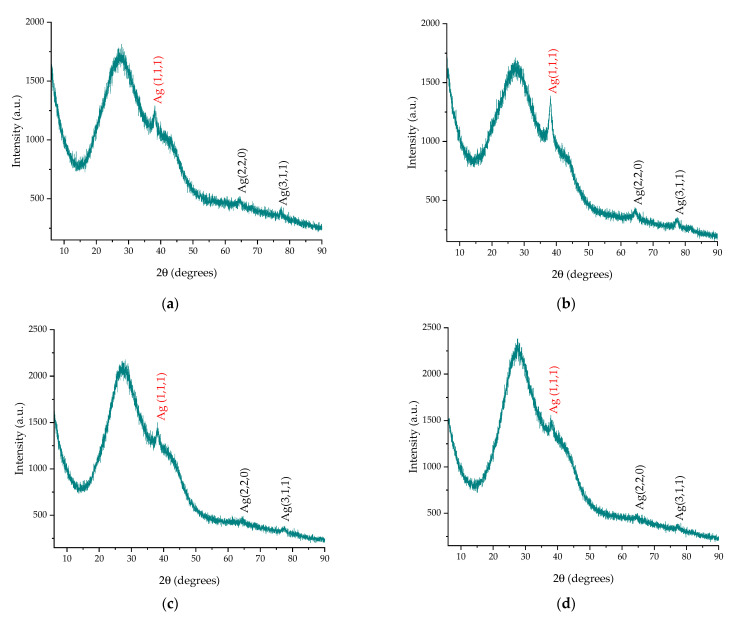
Diffractograms of the phytosynthesized nanoparticles: (**a**) sample P-T-Ag1; (**b**) sample P-T-Ag2; (**c**) sample P-Mw-Ag1; (**d**) sample P-Mw-Ag2; the strongest peaks recorded (corresponding to the (111) diffraction plane, which was further used for determining the crystallite size) are marked with red color.

The X-ray diffraction results provided valuable insights into the crystalline structure of the phytosynthesized nanoparticles. The diffractograms of the samples are presented in Figure 4. In order to perform the analysis, the samples were centrifuged using a DLAB DM0408 (China) laboratory centrifuge at 4000 rpm for a duration of two hours. After removing the supernatant, the samples were deposited on a glass support for analysis. From the analysis of the results, the samples displayed three diffraction peaks at 38°, 64°, and 77°, which corresponded to the diffraction planes (111), (220), and (311), respectively, and the phase could be identified as Ag in a cubic crystalline system, as determined by comparison with the ICDD entry 01-087-0719. Table 3 presents the calculated crystallite dimensions using Equation (2).

**Figure 5:**
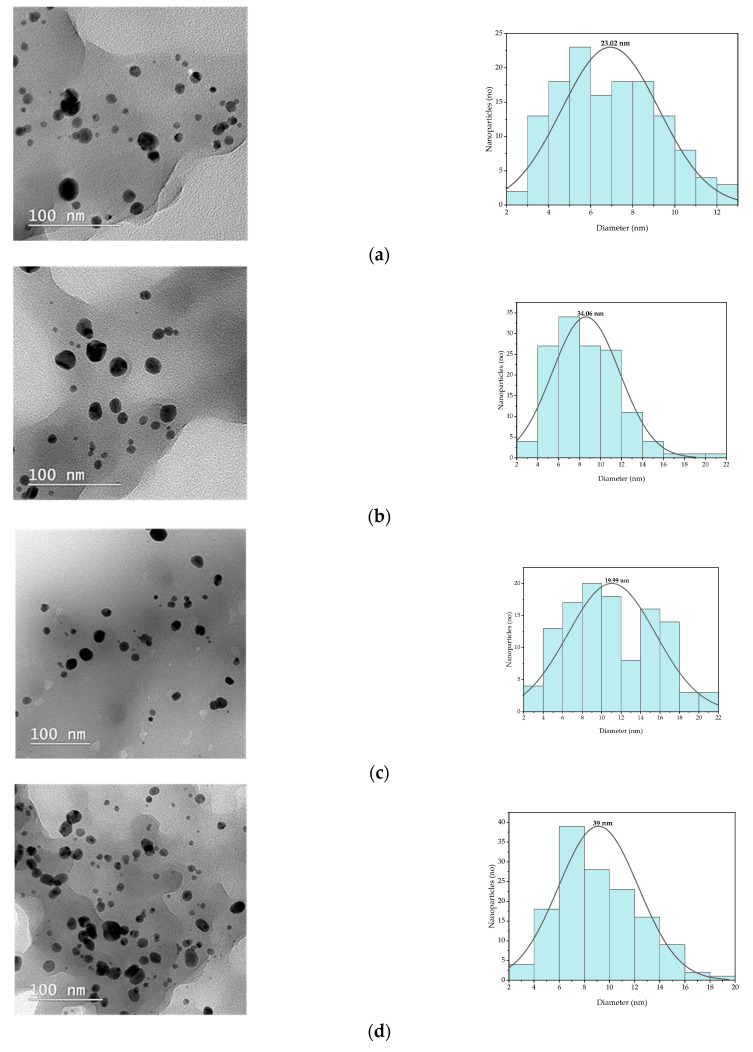
TEM images and size distributions obtained for the phytosynthesized nanoparticles: (**a**) sample P-T-Ag1, (**b**) sample P-T-Ag2, (**c**) sample P-Mw-Ag1, (**d**) sample P-Mw-Ag2.

The TEM images (Figure 5) revealed the presence of spherical or semi-spherical structures with the presence of a small number of other morphologies (triangular, ellipsoidal). The size distribution of the phytosynthesized nanoparticles was determined by 100 measurements/sample, as analyzed with Image J (v. 1.53u, National Institutes of Health, Bethesda, MD, USA) analysis software.

**Figure 6:**
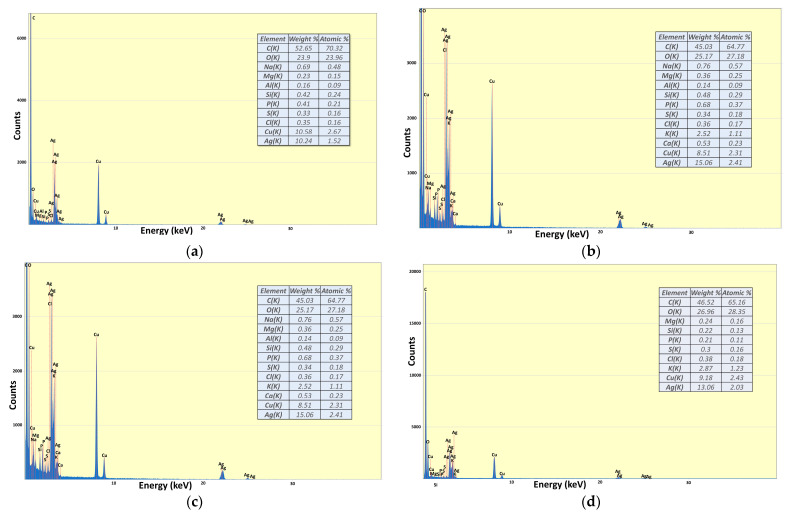
EDX spectra of samples: (**a**) P-T-Ag1; (**b**) P-T-Ag2; (**c**) P-Mw-Ag1; (**d**) P-Mw-Ag2.

EDX spectra (presented in Figure 6) were collected for each sample in order to identify the presence of silver.

**Figure 7:**
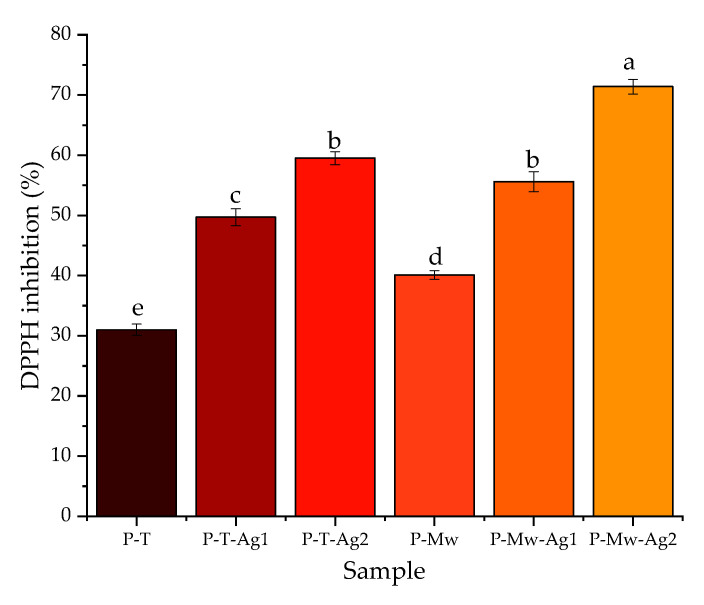
Evaluation of the antioxidant properties of the samples (expressed as DPPH inhibition). Values are means ± SEM, n = 3 per treatment group. Means without a common superscript letter differ (*p* < 0.05), as analyzed by one-way ANOVA and the Tukey test.

**Figure 8:**
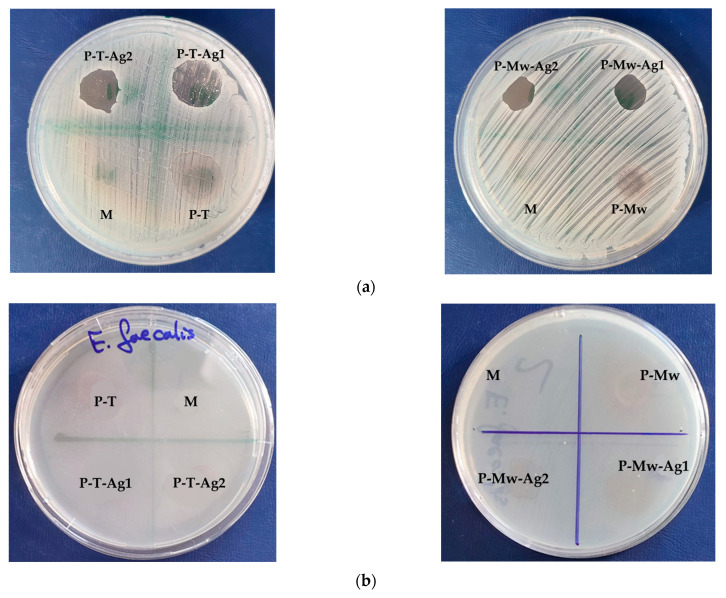
Aspect of qualitative studies (ZOI) results of pomace extracts obtained by classical temperature extraction (P-T) and microwave-assisted extraction (P-Mw) and the nanoparticles phytosynthesized through them (P-T-Ag1, P-T-Ag2, P-Mw-Ag1, and P-Mw-Ag2): (**a**) tests on *Staphylococcus aureus* ATCC 25923; (**b**) tests on *Enterococcus faecalis* ATCC 29212.

**Figure 9:**
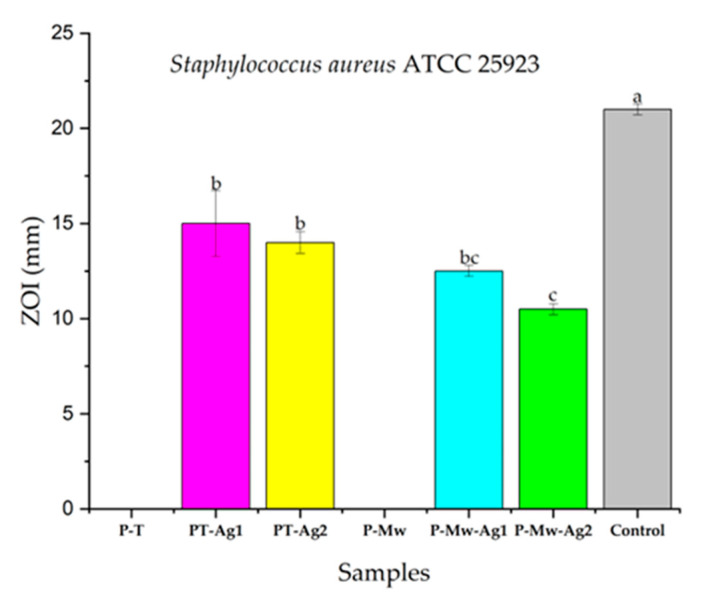
Qualitative results of antimicrobial effects of samples expressed as inhibition zone diameters for *Staphylococcus aureus* ATCC 25923, where the control was clindamycin. Values are means ± SEM, n = 3 per treatment group. Means without a common superscript letter differ (*p* < 0.05), as analyzed by one-way ANOVA and the Tukey test.

**Figure 10:**
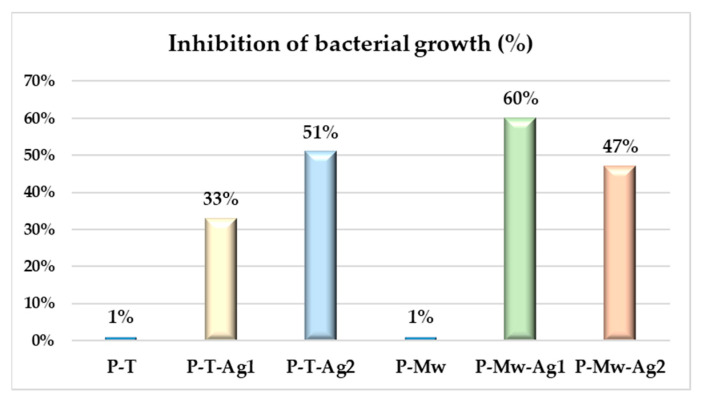
Percentage of bacterial growth inhibition of samples of *Staphylococcus aureus* ATCC 25923.

**Figure 11:**
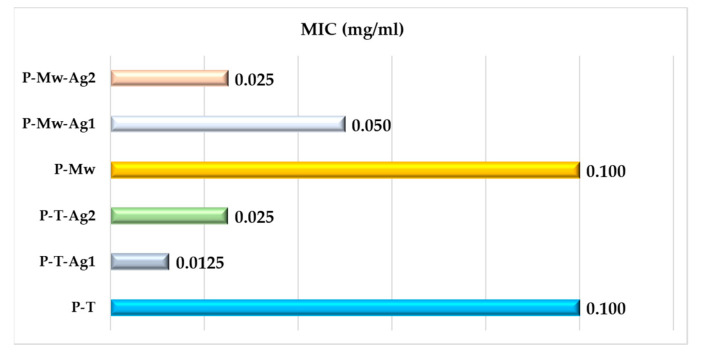
Graphic representation of minimum inhibitory concentration (MIC) obtained by P-Mw series for *Staphylococcus aureus* ATCC 25923.

The results obtained regarding the antioxidant activity exerted by both the extract samples and the related nanoparticle samples are presented in Figure 7, while the results of the antimicrobial assays (qualitative screening, percentage of bacterial growth inhibition, and minimum inhibitory concentration) are presented in Figure 8, Figure 9, Figure 10 and Figure 11.

Considering the absence of any inhibition zone for *E. faecalis,* the other antimicrobial studies were performed exclusively on the *S. aureus* strain.

## 3. Discussion

From Table 1, it can be observed that there was a statistically significant variation in the total phenolics content between the two extraction methods (48.9 and 101 mg/g GAE (dry matter), respectively, showing superior results for the microwave-assisted extraction method). At the same time, the samples exhibited a higher level of total phenolic compounds when comparing the results obtained from the phytochemical tests with the data from the literature [22]. This comparison specifically refers to extracts from the same vine species (*Feteasca Neagră*), although they were obtained by a different extraction method. In the case of the extract obtained by the microwave-assisted method, a much higher content of total phenolic compounds was identified. These results are in accordance with the literature data, which attest to the efficiency of the microwave-assisted extraction method due to the fact that the electromagnetic radiation allows the effective penetration of the solvent into the plant matrix, the solubilization, and/or the breakdown of the components, thus releasing the phenolic compounds of interest from the sample matrix [23].

Using the HPLC technique, 14 compounds were identified in the grape pomace extracts. The experiment was carried out twice, and the findings were reported as average figures and standard deviations (Table 1, chromatograms presented in Figure 2). In the grape pomace extract (obtained by the modern method, P-Mw), tannic acid, gallic acid, vanillic acid, chlorogenic acid, epicatechin, ferulic acid, hyperoside, and rosmarinic acid were detected in a higher concentration in comparison with the extract obtained by classical temperature extraction (P-T). On the other hand, catechin, isoquercetin, and rutin were below the detectable limits in terms of their levels in the case of the P-T sample, as was luteolin-O-glucoside in the case of the P-Mw sample.

Compared to the results presented by Onache et al. [22], our results showed a lower concentration of gallic acid (approx. 10 mg/100 g DW reported in [22]), catechin (approx. 79 mg/100 DW), epicatechin (approx. 70 mg/100 DW), ellagic acid (approx. 3.47 mg/100 g DW), and syringic acid (approx. 48.43 mg/100 g DW), but they showed a higher concentration of chlorogenic acid (under 0.13 mg/100 g DW, reported in [23]), ferulic acid (approx. 0.23 mg/100 g DW), and rutin in the case of the P-Mw extract (under 0.14 mg/100 g DW). Also, the obtained values for isoquercetin and hyperoside were higher than those obtained in the literature data reported by Balea et al. [24], who registered a concentration of approx. 2.5 μg/mL and 0.8 μg/mL, respectively, for the two compounds. The concentrations of tannic acid, vanillic acid, luteolin-O-glucoside, and rosmarinic acid found in grape pomace extracts have not been quantified up to date in similar results according to our knowledge. As a general remark, it must be stated that the phenolic composition of grapevine is strongly influenced by a complex interaction between a series of factors, including the genotype, geographical location of the vineyard, particularly the latitude and altitude, regional seasonal weather fluctuations, or stress conditions [25,26,27]. However, the compositional differences recorded between the two extracts used in the present study represent an important parameter of the phytosynthesis process.

Regarding the phytosynthesis process, Figure 3a,b illustrate the appearance of distinct silver nanoparticle absorption maxima (400–450 nm) for both synthesis methods when utilizing pomace extracts obtained by classical temperature extraction. In the case of the sample P-T-Ag1, the appearance of the absorption maximum specific to silver after 1 h from the initiation of the synthesis can be noted (in a 1:1 ratio of pomace extract: silver nitrate solution). The nanoparticles synthesized with a ratio of 1:2 of extract: silver salt solution displayed much clearer definition in their maximum absorption peaks. In both cases, the peaks tended to shift towards shorter wavelengths, indicating a hypsochromic effect. This effect can be attributed to the gradual reduction in nanoparticle size over time. Additionally, the smallest displacement from the initial maximum position was observed with a shift from 440 to 434 nm in the case of the P-T-Ag2 sample.

In contrast, the absorption maxima varied for the series of phytosynthesized nanostructures using the pomace extract obtained by microwave-assisted extraction, as shown in Figure 4a,b. The P-Mw-Ag1 sample exhibited a bathochromic shift from 435 nm to 443 nm, suggesting an agglomeration of nanoparticles. For the P-Mw-Ag2 sample, a better-defined absorption maximum can be observed as well as the presence of a hypsochromic effect from 444 nm to 433 nm. In this case, the lower concentration of extract (with a high phenolic content, as previously presented) managed to efficiently reduce the double ratio of silver nitrate, and the phytosynthesis process was much more stable and occurred without the appearance of nanoparticle agglomeration. 

In conclusion, the phytosynthesis process of silver nanoparticles was confirmed for all the synthesis procedures applied in a relatively short time, i.e., 96 h, without any agglomeration tendency in the case of P-T-Ag1, P-T-Ag2 and P-Mw-Ag2 and with low agglomeration in the case of P-Mw-Ag1.

In order to confirm these findings, X-ray diffraction analysis was applied in order to gain insights into the crystalline nature of the materials as well as their crystallite size (as determined from the strongest peak’s parameters, corresponding to the diffraction plane (111)). From the XRD data, it can be observed that in the case of using a ratio of 1:1 *v*/*v* (grape pomace extract: silver nitrate) for the phytosynthesis of nanoparticles, smaller crystallite sizes were obtained (5.14 nm for P-T-Ag1 and 4.58 nm for P-Mw-Ag1) compared to the synthesis process that involved a ratio of 1:2 *v*/*v* (grape pomace extract: silver nitrate), for which larger crystallite sizes were obtained (8.64 nm for P-T-Ag2 and 9.64 nm for P-Mw-Ag2). At the same time, it is notable that the phytosynthesis process was significantly affected by the overall phenolic content. The nanoparticles produced using the extract obtained by the microwave-assisted extraction method exhibited a smaller crystallite size compared with the extract obtained by classical temperature extraction.

The TEM images (Figure 5) revealed the presence of mostly spherical or semi-spherical structures with the presence of a small number of other morphologies (triangular and ellipsoidal). Thus, it can be stated that using the described method, silver nanoparticles with a relative uniform morphology were obtained, compared with the literature data, in which much more diverse morphologies were obtained [18]. The size distribution of the phytosynthesized nanoparticles was determined by direct measurement of over 100 nanoparticles/sample. From the distribution curve of the nanoparticle size, the nanoparticles phytosynthesized using the extracts obtained by both methods exhibited average diameters of between 20 and 39 nm. It can be observed that the nanoparticles synthesized in a 1:1 *v*/*v* ratio (extract: silver nitrate) exhibited smaller dimensions (average diameter of 19.99 for sample P-Mw-Ag1 and of approx. 23 nm for P-T-Ag1, respectively), and, at the same time, the nanoparticles tended to have larger sizes when they were synthesized with a higher metal precursor concentration, i.e., 34 nm in the case of P-T-Ag2 and 39 nm for the P-Mw-Ag2 sample, which were in excellent agreement with the XRD data. The synthesis of silver nanoparticles was reconfirmed by the EDX spectra in all instances, and the results are displayed in Figure 6. Although the EDX analysis did not generally offer a clear image of the entire sample composition, from the presented results, it can be observed that silver was the most abundant element (after C and O, originating from the phytoconstituent composition, and Cu, originating from the copper grids used for analysis).

**Figure 12:**
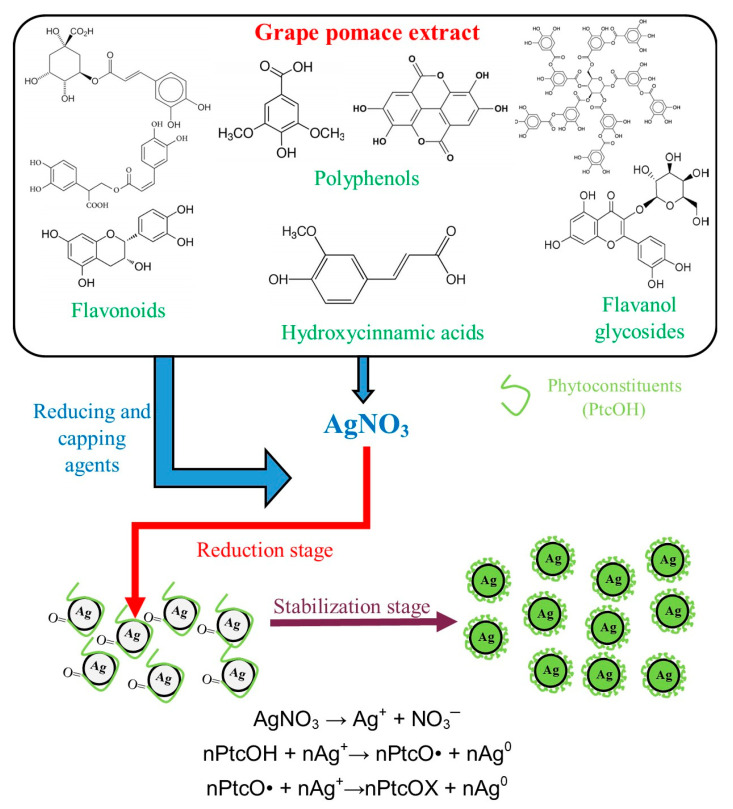
Proposed mechanism and reactions involved in the phytosynthesis of silver nanoparticles using grape pomace extracts and the reactions involved.

Considering the obtained results as well as our previously published works, a mechanism for the phytosynthesis of silver nanoparticles using grape pomace extracts can be proposed (Figure 12).

The DPPH antioxidant assay revealed an increase in the antioxidant potential of the phytosynthesized nanoparticles compared with the parent extracts, as well as a superior antioxidant potential of the extract obtained by the microwave-assisted method (compared with the temperature extraction method). At the same time, the antioxidant potential was found to vary in a concentration-dependent manner with the silver content. Expressed as quercitin equivalents, the results were found to vary from approx. 0.66 to approx. 3.64 mg QE/L (temperature extraction vs. microwave-assisted extraction). For the same extraction procedure, the silver concentration dependency of the antioxidant capacity of the phytosynthesized nanoparticles can be expressed (as quercetin equivalents) as follows: approx. 2.03 mg QE/L (P-T-Ag1) vs. 2.76 mg QE/L (P-T-Ag2) and approx. 2.47 mg QE/L (P-Mw-Ag1) vs. 3.63 mg QE/L (P-Mw-Ag2), respectively. These findings are in good concordance with literature data, with several authors presenting the antioxidant potential of grapevine wastes extracts, as determined using DPPH assays [28,29]. A direct comparison with the literature data regarding antioxidant potential is often a difficult endeavor considering the differences in the extraction procedure, antioxidant assays performed, standard antioxidant used, and reporting method. Other authors have observed a dose-dependent increase in the DPPH inhibition percentage using grape pomace extracts obtained by different methods, reaching an inhibition of over 70% for a 7.5 mg/L extract concentration, which is superior to the antioxidant potential of ascorbic acid (used as a standard) at similar concentrations [30]. Using different solvents (methanol, ethyl acetate, and water), Murthy et al. [31] evaluated the antioxidant potential of grape pomace extracts at different concentrations, as determined by a DPPH assay, obtaining inhibitions of from 9.1 to 42.1% (at a concentration of 50 mg/kg) in a solvent-dependent manner. At the same time, Zhu et al. [32] identified a direct correlation between the total polyphenol content and the DPPH scavenging ability of grape pomace extracts; this correlation could also explain the differences recorded in our study regarding the antioxidant potential of the two extracts. Also, phytosynthesized silver nanoparticles have commonly been found by other authors to significantly increase the antioxidant activity of the parent extract [33] in a silver-concentration-dependent manner [34], which is in good agreement with the results of the present study.

The results are of particular Interest, as the search for new antioxidant agents represents a main research area. Whether we are talking about metabolism-resultant free radicals, those originating from environmental contamination, or those generated by ionizing radiation, free radicals can be linked to different severe illnesses (including Parkinson’s and Alzheimer’s diseases, atherosclerosis, and cancer) [35]. Nano-antioxidants (including phytosynthesized nanoparticles) can be used to mitigate free radical production and action, thus offering important alternatives for the treatment of free-radical-associated diseases [35]. However, further cytotoxicity assays are necessary in order to validate them in these types of applications.

After conducting the qualitative screening, the antimicrobial effectiveness of the samples against the *Staphylococcus aureus* ATCC 25923 strain was observed by examining the presence of a growth inhibition halo. This was observed in case of P-T-Ag1, P-T-Ag2, P-Mw-Ag1, and P-Mw-Ag2. The sample P-T-Ag1 exhibited the largest diameter of the inhibition zone (ZOI), measuring 15 mm, while the smallest diameter was observed in the case of the P-Mw-Ag2 sample, measuring 10 mm. The aspect of the qualitative studies (Petri dishes) is presented in Figure 8, and the results are presented in Figure 9. For the *Enterococcus faecalis* ATCC 29212 strain, none of the samples led to the apparition of an inhibition halo.

The results obtained could seem, at first, surprising, as both strains are Gram-positive bacteria, which are commonly used in antimicrobial assays and are usually reported to be inhibited by natural extracts and/or silver nanoparticles. However, the characteristics of each bacterium should be also considered. The primary components of the cell wall in Gram-positive bacteria are represented by three main constituents: peptidoglycan, anionic polymers (teichoic acids and cell wall polysaccharides), and wall-anchored proteins, with the overall thickness of the cell wall being around 20–35 nm [36]. In contrast, the thickness of the cell wall of *E. faecalis* is around 40 nm [37]. The chemical structures of *S. aureus* and *E. faecalis* also differ in their bridge structure; in *S. aureus*, the bridge consists of pentaglycine (short-length and extensively cross-linked) [38], while in *E. faecalis*, the peptide cross-bridge comprises 2–3 L-Ala (L-alanine) residues [39]. The cross-linking in the *E. faecalis* cell wall is approximately 50% (due to the short peptidoglycan bridge), whereas in *S. aureus*, it is around 85% (as a result of the pentaglycine cross-bridge) [40,41]. In addition, the literature data suggest that silver nanoparticles could damage lipoteichoic acid (LTA) (a particularly important component of the *S. aureus* cell envelope) [42]. The nanoparticle-induced damage also hinders the peptidoglycan cross-linking process, making the bacteria more vulnerable to the effects of the nanostructure. This could potentially be one of the reasons why the samples exhibited antimicrobial activity exclusively for *S. aureus,* suggesting a direct correlation between the cell wall cross-linking and the sensitivity to the phytosynthesized silver nanoparticles.

The literature data also provide similar results regarding the different sensitivities of Gram-positive bacteria to the antimicrobials tested. For example, Rosato et al. [43] obtained significant differences between the minimum inhibitory concentrations of both a synthetic antimicrobial agent (gentamicin) and *Mentha piperita* essential oil against *E. faecalis* ATCC 29212 (the same strain used in the present study) and *S. aureus* ATCC 29213 (a similar non-resistant strain to the one used in the present study), with an MIC value twice as high for the essential oil (against *E. faecalis* compared with *S. aureus)* and sixteen times higher for the synthetic drug. In another study, Perveen et al. [44] identified significant differences between the inhibition zones recorded for the synthetic drug doxycycline against the *S. aureus* and *E. faecalis* strains (the *S. aureus* strain being more sensitive); the authors also evaluated several *Phoenix dactylifera* L. leaf and pit extracts against the two strains, concluding that the extracts were not active against the *E. faecalis* strain, while different inhibition zones were recorded against the *S. aureus* strain (the diameter of the inhibition zones depended on the variety, solvent, and plant part used).

The recorded differences can also be explained by several other factors. *E. faecalis* possesses efflux pumps that can actively remove toxic substances from within the cell. These pumps can contribute to the bacterium’s resistance to various compounds, including those present in natural extracts, as well as to silver nanoparticles [45]. Other factors that could also influence the sensitivity of different strains to the tested materials are genetic variation and metabolic pathways [46].

The antimicrobial action mechanism of AgNPs involve several processes:

- Cell membrane damage. AgNPs can interact with the bacterial cell membrane, leading to a disruption of the membrane’s integrity. This disruption causes a leakage of intracellular components, loss of membrane potential, and eventual cell death. AgNPs can penetrate the bacterial cell membrane, disrupting its structure and function. This penetration disturbs membrane-bound proteins and enzymes, affecting essential cellular processes [47,48].
- Generation of reactive oxygen species (ROS). AgNPs can generate ROS, such as superoxide radicals (O_2_^−^), hydrogen peroxide (H_2_O_2_), and hydroxyl radicals (OH^−^). These ROS induce oxidative stress within bacterial cells, damaging proteins, lipids, and DNA. ROS-mediated damage to cellular components disrupts bacterial metabolism, impairs cellular respiration, and leads to cell death [48].
- Inhibition of DNA replication and protein synthesis. AgNPs can bind to bacterial DNA, interfering with DNA replication and transcription processes. This binding disrupts DNA integrity, leading to DNA strand breaks, replication errors, and inhibition of protein synthesis. AgNPs can also interact with ribosomal subunits, inhibiting protein synthesis and impairing bacterial growth and proliferation [48,49].
- Disruption of electron transport chain (ETC). AgNPs can interfere with the electron transport chain (ETC) in bacterial cells, disrupting ATP synthesis and energy metabolism. This disruption leads to a depletion of cellular energy stores, metabolic dysfunction, and, ultimately, cell death. The inhibition of ETC components such as cytochromes and other electron carriers by AgNPs impairs bacterial respiration and ATP production [48].
- Induction of apoptosis-like cell death. AgNPs can trigger apoptosis-like cell-death pathways in bacteria, leading to programmed cell death. This process involves the activation of caspase-like enzymes, DNA fragmentation, and morphological changes characteristic of apoptosis. Apoptosis-like cell death induced by AgNPs prevents the release of inflammatory mediators and reduces the risk of inflammatory responses associated with bacterial infections [50].

All these effects are enhanced by the presence of phytoconstituent capping agents (including phenolic compounds, flavonoids, terpenoids, or alkaloids), which could also act as potent antimicrobial agents through the disruption of cell membrane integrity, the inhibition of enzyme activity, the disruption of biofilm formation, interference with quorum sensing (QS), or the induction of oxidative stress [51,52,53,54].

Following the quantitative test (MIC), an efficiency against *S. aureus* was observed. A graphic representation of the percentage of bacterial growth inhibition is presented in Figure 10, while the MIC values are given in Figure 11. The final antimicrobial properties were not significantly affected by the variations in the dimensions of the nanoparticles. The results obtained are superior to those reported by Parvekar et al. [55] regarding the MIC value recorded for commercial AgNPs (0.625 mg/mL), underlining the important contribution of the phytoconstituents to the final biological properties of the materials. Compared with the literature data regarding phytosynthesized silver nanoparticles, the obtained results revealed slightly lower minimal inhibitory concentrations (although comparable) than the values obtained by Giri et al. [56] on the application of silver nanoparticles obtained using *Eugenia roxburghii* DC extract.

Sample P-Mw-Ag1 (having an average nanoparticle diameter of 19.99 nm) showed the highest efficiency, inhibiting 60% of bacterial growth at a concentration of 0.05 mg/mL. Sample P-T-Ag1 (23.02 nm) showed the lowest efficiency, inhibiting 33% of the growth of the *S. aureus* strain at a concentration of 0.0125 mg/mL. Samples P-T-Ag2 (34 nm) and P-Mw-Ag2 (39 nm) showed almost similar efficiencies, inhibiting 51% and 47% of the bacterial growth at a product concentration of 0.025 mg/mL. 

The results of the antimicrobial assays suggest a possible selectivity of these types of phytosynthesized nanoparticles towards *S. aureus*, which would allow their proposal as tailored antimicrobials against specific pathogens. Further studies are necessary to determine the exact cause of the selectivity.

## 4. Materials and Methods

### 4.1. Collection and Processing of Vegetal Waste Material

**Figure 13:**
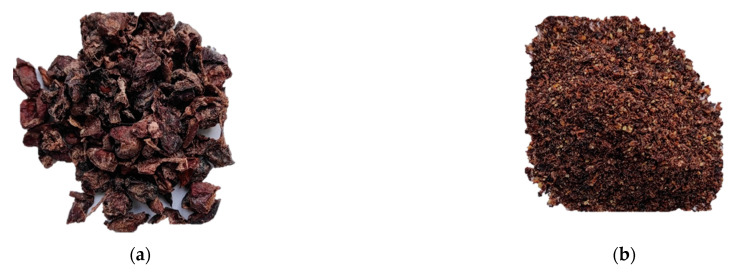
(**a**) The aspect of grape pomace subjected to the drying process; (**b**) dried and shredded samples of grape pomace in order to carry out the experiments.

The grape pomace (originating from the native genotype *Fetească Neagră* 6 Șt.; maintainer: National Research and Development Institute for Biotechnology in Horticulture, Stefanesti, registration certificate number 1698/2005) samples were collected immediately after pressing the grapes. To avoid oxidation, the samples were stored in vacuum bags at −20 °C. Before starting the experiments, the samples were subjected to a drying procedure using a JW-MW-6 KW microwave oven at a temperature of 65 °C (Figure 13a). Finally, the dried pomace samples were shredded using a laboratory mill (Figure 13b).

### 4.2. Preparation and Characterization of Grape Pomace (P) Extracts and Nanoparticle Phytosynthesis

The success of the extraction procedure of bioactive compounds depends on a series of factors, including the type of vegetal material, temperature, extraction time, and solvent used. In order to obtain natural extracts that could be used to phytosynthesize silver nanoparticles (the final goal of the study), these parameters were carefully taken into account. Two different extraction methods were applied to obtain extracts from grapevine wastes, with plant material being used for both methods at a solvent ratio of 2:10 *w*/*v*; the solvent of choice was represented by a hydroalcoholic solvent (the ratio distilled water to ethanol being, in both cases, 1:1 *v*/*v*). The distilled water was obtained in the laboratory using Liston A1210 water still (Liston, Moscow, Russia), and the ethanol used as a solvent was of reactive quality (Ethanol 96% Carl ROTH Gmbh, Karlsruhe, Germany).

Briefly, the two extraction methods can be presented as follows:

- Classical temperature extraction (T). The grapevine waste material was mixed with the solvent, and the extraction was carried out using a BOV-T70C oven (Biobase, Shandong, China) with an extraction time of 60 min and at a temperature of 60 °C; the obtained extract was encoded as P-T.
- Microwave-assisted extraction (Mw). The weighed vegetal material was placed in Teflon tubes, to which the solvent (in the previously presented ratio) was added. The extraction was performed using an Ethos Easy Advanced Microwave Digestion System (Milestone Srl, Sorisole, Italy). The extraction parameters were as follows: extraction time: 30 min, extraction temperature: 60 °C, maximum microwave power: 1000 W; the obtained extract was encoded P-Mw.

After the extraction processes were completed, the samples underwent filtration using filter paper and were then stored in a refrigerator for further experiments.

The phytosynthesis process of nanoparticles relies on the presence of phytoconstituents in the natural extracts. To quantify the total phenolic content of the acquired extracts, the Folin–Ciocâlteu spectrophotometric method was used. 

For the determination of the total phenolic content, the extracts (P-T and P-Mw) were mixed with the Folin–Ciocâlteu reagent (Merck KGaA, Darmstadt, Germany), and a specific volume of sodium carbonate solution (Merck KGaA, Darmstadt, Germany) was introduced to each test tube. After 60 min, the optical density of the samples was determined at 765 nm using a Rigol Ultra 3660 UV–Vis spectrophotometer (Rigol Technologies, Beijing, China). The registered results were compared with a standard curve of gallic acid. Thus, the total phenolic content was expressed as mg/g of gallic acid equivalents (GAE), applying the following formula:

(1) $$C_{\mathrm{TP}}=c\;\times \;\frac{V}{m}$$

where C_TP_ is the total content of phenolic compounds (µg/g) expressed as gallic acid equivalents (GAE), c represents the concentration of gallic acid obtained from the calibration curve in µg/mL, V is the volume of the extract in mL, and m is the extract mass used in grams. Three determinations were performed in triplicate, and the results were presented as mean ± the standard error of the mean.

The quantification of the selected components was carried out using high-performance liquid chromatography with a diode array detector (HPLC-DAD, Rigol Technologies Inc., Beijing, China) using a Kinetex EVO C18 column (150 × 4.6 mm, particle size = 5 μm) at a temperature of 30 °C. The solvent mixtures applied were as follows: solvent A consisted of H_2_O with 0.1% trifluoroacetic acid (TFA), and solvent B comprised acetonitrile (ACN) with 0.1% TFA. The flow rate was 1 mL/min, with a gradient elution ranging from 2% to 100% B. The duration of the process was set at 60 min, and detection occurred at a wavelength of 280 nm. The reference compounds (tannic acid, gallic acid, catechin, vanillic acid, chlorogenic acid, syringic acid, epicatechin, ferulic acid, ellagic acid, isoquercetin, rutin, luteolin-O-glucoside, hyperoside, and rosmarinic acid) were reagent-grade (Merck KGaA, Darmstadt, Germany) and were used without further purification. These stock solutions were prepared with a concentration of 1000 µg/mL. To create the calibration curves, concentrations ranging from 10 to 400 µg/mL were used.

The content of bioactive compounds present in extracts contributes to the reduction of metal ions, leading to the formation of nanomaterials. For the phytosynthesis, each extract was mixed with a 10^−3^ M silver nitrate solution (Chimreactiv, Bucharest, Romania). In order to study the influence of the natural-extract-to-metal-salt ratios, two experimental conditions were selected (1:1 and 1:2 *v*/*v* extract: metal salt ratio). The phytosynthesis process was initiated with the mixture of the two components, and the reaction was carried out under ambient conditions. The formation of the nanoparticles could be visually monitored by observing the apparition of the distinct dark red color of the silver nanoparticles [57]. Table 4 displays the encoding of the nanoparticle samples.

The phytosynthesized nanoparticles (with the encodings presented in Table 4) were studied by different analytical methods of characterization in order to obtain the most relevant data about their formation, nature, and structure.

The first analytical method applied (used for monitoring the formation of the NPs) was UV–Vis spectrometry. UV–Vis spectra of the samples were measured at various time intervals (1 h, 21 h, 48 h, 72 h, and 96 h) in the wavelength range of 300–700 nm using an Ultra 3660 UV–Vis spectrophotometer (Rigol Technologies Inc., Beijing, China). 

The X-ray diffractograms of the samples were acquired in the range of 5–85 degrees (2θ) using a 9 kW Rigaku SmartLab diffractometer (Rigaku Corporation, Tokyo, Japan), operating with the following parameters: 45 kV, 200 mA, Cu_Kα_ radiation (1.54059 Å), and parallel beam configuration (2θ/θ scan mode). The obtained diffractograms were analyzed using the Rigaku PDXL 2 data analysis software (ver. 2.7.2.0, Rigaku Corporation, Tokyo, Japan), and the identification of the phases present in the samples was carried out by comparison with entries from the ICDD (International Center for Diffraction Data) database. The crystallite size of the nanoparticles was calculated using the Debye–Scherrer equation, which is as follows:

(2) $$Dp=\frac{\left(K\times \;\lambda \right)\;}{\left(\beta \times \cos\theta \right)}$$

where D*p* represents the mean crystallite size, K represents the Scherrer constant (K = 0.94), λ is the wavelength (1.54059 Å), β is the full width at half maximum, and θ represents the Bragg angle.

The size and shape of the phytosynthesized nanostructures were determined through the application of transmission electron microscopy (TEM). TEM micrographs were obtained using a Tecnai F20 G² TWIN Cryo-TEM device (FEI, Hillsboro, OR, USA) at an acceleration voltage of 200 kV. Carbon film on copper grids was used (Ted Pella Inc., Redding, CA, USA). An X-MaxN 80T detector (Oxford Instruments, Tubney Woods, UK) was used to obtain the EDX spectra. The size distribution was determined by direct measurement of over 100 nanoparticles from the TEM images with the use of the Image J image analysis software (v. 1.53u, National Institutes of Health, Bethesda, MD, USA).

### 4.3. Evaluation of Antioxidant and Antimicrobial Properties

The evaluation of the antioxidant activity was carried out both for the obtained extracts and for the synthesized nanoparticles using the DPPH method. The principle of this technique consists of the reduction of a free radical—DPPH (purple appearance)—by antioxidant substances. The samples were mixed 1:1 *v*/*v* with DPPH reagent (Sigma Aldrich, St. Louis, MO, USA), and their absorbances were recorded at a 517 nm wavelength using Rigol Ultra 3660 UV–Vis spectrometer (Rigol Technologies Inc., Beijing, China). The antioxidant activity of the samples is directly proportional to the DPPH radical inhibition percentage. The antioxidant activity was calculated based on the following formula:

(3) $$\mathrm{AA}\%=\frac{A\;517\mathrm{nm}\;\left(\mathrm{blank}\right)-A\;517\mathrm{nm}\;\left(\mathrm{sample}\right)\;}{A\;517\mathrm{nm}\;\left(\mathrm{blank}\right)}\times 100$$

where AA% is the antioxidant activity of the samples, A_517nm_(blank) is the absorbance measured at 517 for the simple DPPH solution, and A_517nm_(sample) is the absorbance measured at 517 for the mixture of DPPH solution and sample.

Three determinations were performed for each sample, and the results were presented as the average of the determinations ± the standard error of the mean.

**Figure 14:**
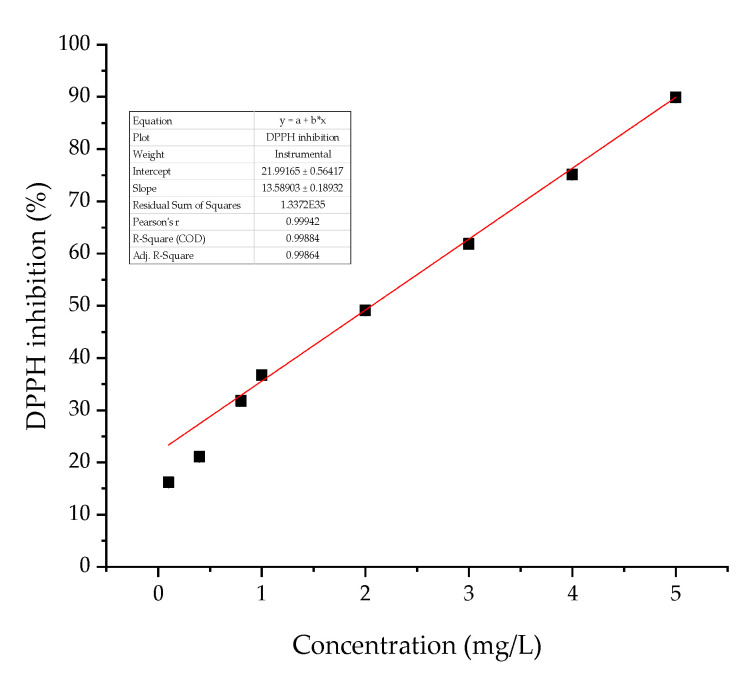
Calibration curve for the antioxidant potential of quercetin.

Furthermore, in order to provide a clear image of the antioxidant properties of the extracts and the phytosynthesized nanoparticles, quercetin (Sigma Aldrich, St. Louis, MO, USA) was used to construct a calibration curve (in the range 0.1–5 mg/L). DPPH inhibition percentages were plotted against the concentration, and the linear fit function was applied; the calibration equation calculated was y = 21.991 + 13.589 × (R^2^ = 0.9986) (Figure 14). The results obtained for the extracts were reported as mg quercetin equivalents (mgQE)/L using the above-mentioned equation.

The antimicrobial efficiency of the phytosynthesized nanoparticles was assessed against different strains, which were chosen based on their pathogenic nature. The tests were carried out on the following Gram-positive bacteria obtained from the microbiological collection of the National Institute for Research & Development in Chemistry and Petrochemistry, ICECHIM, Bucharest: *Staphylococcus aureus* ATCC 25923 and *Escherichia coli* ATCC 25922.

In order to evaluate the antimicrobial potential of the nanoparticles, the diffusion test was applied, along with broth serial dilutions for the determination of minimum inhibitory concentrations (MICs). Aliquots of 20 µL nanoparticles were distributed in spots on agar media previously inoculated with fresh bacterial inoculum. For the qualitative tests, the plates were incubated at 37 °C for 24 h, and the growth inhibition zones (ZOI) were measured. The diameter of the inhibition zones was correlated with the antimicrobial efficiency of the products. Simultaneously, a positive control represented by microbial growth in the absence of the product and a negative control represented by an antibiotic were prepared. The following antibiotics were tested: clindamycin (2 µg) for *S. aureus* and gentamicin (10 µg) for *E. coli.*

Serial dilutions were employed to assess the MIC in Mueller–Hinton broth (Sharlau, Spain, g/L: 17.5, casein hydrolysate; 2, beef extract; 1.5, starch). The concentrations ranged between 50 and 1.56%. A volume of 10 µL of fresh inoculum of 0.5 McFarland standard density was added to each well in 96-well plates. Positive and negative controls were also prepared. The plates were incubated at 37 °C for 24 h, and their optical density was further determined using a SpectraMax Id5 plate reader (Molecular Devices, CA, USA) at a 600 nm wavelength. The MICs were evaluated as the lowest concentration that inhibited the bacterial growth, and the ODs of the sample and the positive control were compared in order to assess the growth inhibition (%).

**Scheme 1:**
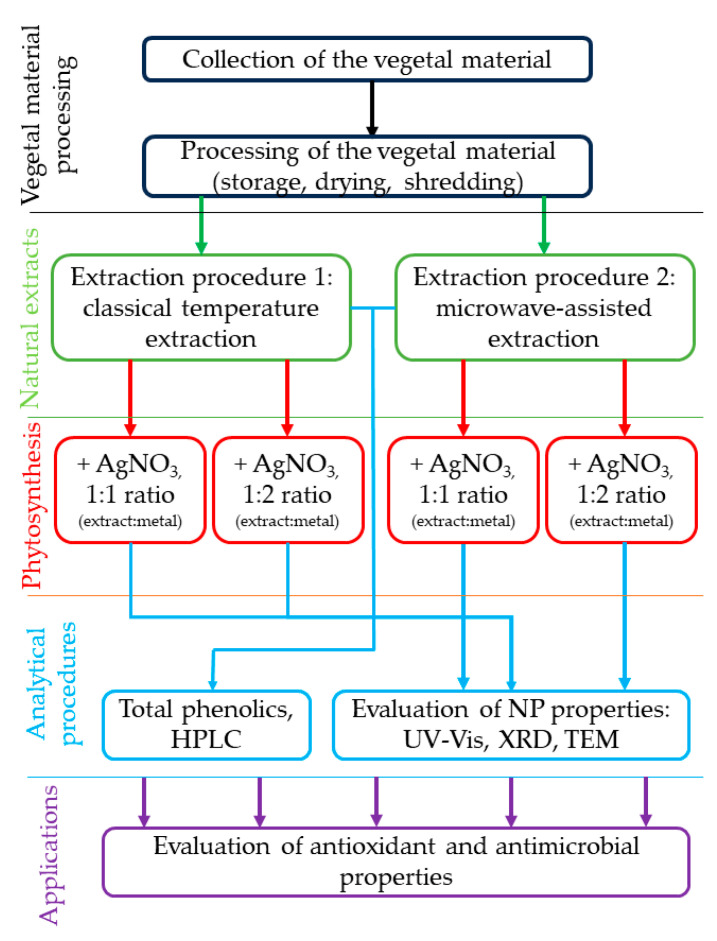
Experimental design applied in the present study.

The experimental design is graphically presented in Scheme 1.

### 4.4. Statistical Interpretation and Data Representation

The analyses were performed by carrying out multiple parallel determinations (as mentioned for each method), and the data obtained were analyzed for statistical significance using analysis of variance (one-way ANOVA) and Tukey’s test to determine significant differences between means. Significant differences were set at *p* ≤ 0.05. 

Graphical representations were constructed using the OriginPro 2018 Data Analysis and Graphing Software (v. 95E, OriginLab Corporation, Northampton, MA, USA).

## 5. Conclusions

In conclusion, the presented green approach demonstrates the potential of grape pomace wastes originating from a Romanian native variety (*Fetească Neagră* 6 Șt.) to be used for the phytosynthesis of silver nanoparticles. The current work not only establishes the potential of this particular type of grapevine industry waste for application in nanotechnology but also evaluates the influence of the extraction method and the extract-to-silver-salt ratio on the final morpho-structural properties of the silver nanoparticles. According to the TEM and XRD findings, the nanoparticles with the smallest dimensions were achieved by using the extract obtained via the microwave-assisted technique with a 1:1 *v*/*v* ratio of extract to silver nitrate. The evaluation of the antioxidant properties revealed an increase in DPPH inhibition upon NP formation (in a silver-content-dependent manner) compared with the parent extracts as well as a significant difference in terms of the antioxidant potential between the extracts (superior results being obtained for the microwave-assisted extraction method), which was correlated with the extract composition (particularly with the total phenolics content). The evaluation of the antimicrobial properties revealed a selectivity of the tested materials, with all experimental variants exhibiting good antimicrobial properties against the *S. aureus* strain and without any activity being recorded against the *E. faecalis* strain. The obtained results support the application of silver nanoparticles phytosynthesized using grape pomace wastes as selective antibacterial agents as well as in formulations designed to control oxidative stress. However, further studies are needed in order optimize the therapeutic efficacy of the NPs and to evaluate the toxicity of the phytosynthesized nanoparticles.

## Figures and Tables

**Table 1:** The total phenolic content of the analyzed extracts and concentration of target compounds obtained by HPLC ^1^.

Compound	Retention Time (min.)	Extract P-T	Extract P-Mw
Total phenolic content (mg GAE/g dry weight)	-	48.9 ± 0.00508 ^b^	101 ± 0.008 ^a^
Tannic acid (mg/L)	2.6	13.3 ± 0.119 ^b^	15.1 ± 0.135 ^a^
Gallic acid (mg/L)	5.587	1.37 ± 0.0121 ^b^	1.68 ± 0.015 ^a^
Catechin (mg/L)	13.987	N.D.	19.7 ± 0.177 ^a^
Vanillic acid (mg/L)	14.207	13.3 ± 0.118 ^b^	26.6 ± 0.238 ^a^
Chlorogenic acid (mg/L)	15.7	5.18 ± 0.0462 ^b^	7.02 ± 0.0629 ^a^
Syringic acid (mg/L)	15.92	14.8 ± 0.132 ^a^	13.4 ± 0.12 ^b^
Epicatechin (mg/L)	16.893	48.6 ± 0.434 ^b^	100 ± 0.897 ^a^
Ferulic acid (mg/L)	20.743	5.37 ± 0.0479 ^b^	8.15 ± 0.0727 ^a^
Ellagic acid (mg/L)	26.387	3.47 ± 0.0312^a^	1.16 ± 0.0104 ^b^
Isoquercetin (mg/L)	30.813	N.D.	57.2 ± 0.511 ^a^
Rutin (mg/L)	30.953	N.D.	25 ± 0.223 ^a^
Luteolin-O-glucoside (mg/L)	31.237	158 ± 1.41 ^a^	N.D.
Hyperoside (mg/L)	31.423	12.9 ± 0.115 ^b^	25.1 ± 0.224 ^a^
Rosmarinic acid (mg/L)	33.993	0.559 ± 0.0052 ^b^	1.11 ± 0.00981 ^a^

^1^ Values are means ± SEM, n = 3 per treatment group (three independent determinations). Means in a row without a common superscript letter differ (*p* < 0.05), as analyzed by one-way ANOVA and the Tukey test; N.D.—not detected.

**Table 2:** Evaluation of nanoparticle formation over time using extracts of grape pomace obtained by classical temperature extraction and microwave-assisted extraction (Pmax—characteristic peak maximum position).

Time	NPs Obtained by Classical Temperature Pomace Extract		NPs Obtained by Microwave-Assisted Pomace Extract	
	P-T-Ag1_Pmax_	P-T-Ag2_Pmax_	P-Mw-Ag1_Pmax_	P-Mw-Ag2_Pmax_
1 h	448	-	-	-
21 h	446	440	435	444
48 h	446	436	443	442
72 h	442	436	441	441
96 h	439	434	443	433

**Table 3:** The positions of the diffraction maxima and the crystallite sizes of the samples under analysis that were determined based on Equation (2).

Sample	Peak Position (1,1,1), °	Peak Position (2,2,0), °	Peak Position (3,1,1), °	FWHM, ° ^1^	Crystallite Size (nm) ^1^
P-T-Ag1	38.17	64.30	77.16	1.7084	5.14
P-T-Ag2	38.25	64.43	77.32	1.0163	8.64
P-Mw-Ag1	38.09	64.57	77.69	1.9154	4.58
P-Mw-Ag2	38.09	64.12	77.25	0.912	9.62

^1^ Data for the (111) diffraction plane.

**Table 4:** Encoding of phytosynthesized silver nanoparticles.

Type of Extract Used	NPs Encoding	
	Extract:AgNO_3_ Ratio: 1:1 (*v*/*v*)	Extract:AgNO_3_ Ratio: 1:2 (*v*/*v*)
Classical temperature grape pomace extract (P-T)	P-T-Ag1	P-T-Ag2
Microwave-assisted grape pomace extract (P-Mw)	P-Mw-Ag1	P-Mw-Ag2

## Data Availability

The data presented in this study are available on request from the corresponding authors.
